# Desmoglein 2 Depletion Leads to Increased Migration and Upregulation of the Chemoattractant Secretoneurin in Melanoma Cells

**DOI:** 10.1371/journal.pone.0089491

**Published:** 2014-02-18

**Authors:** Wiebke K. Peitsch, Yvette Doerflinger, Reiner Fischer-Colbrie, Volker Huck, Alexander T. Bauer, Jochen Utikal, Sergij Goerdt, Stefan W. Schneider

**Affiliations:** 1 Department of Dermatology, University Medical Center Mannheim, Heidelberg University, Mannheim, Germany; 2 Helmholtz Group for Cell Biology, German Cancer Research Center (DKFZ), Heidelberg, Germany; 3 Department of Pharmacology, Innsbruck Medical University, Innsbruck, Austria; 4 Skin Cancer Unit, German Cancer Research Center (DKFZ), Heidelberg, Germany; University Hospital Hamburg-Eppendorf, Germany

## Abstract

During development and progression of malignant melanoma, an important role has been attributed to alterations of cell-cell adhesions, in particular, to a “cadherin switch” from E- to N-cadherin. We have previously shown that a subtype of melanoma cells express the desmosomal cadherin desmoglein 2 as non-junction-bound cell surface protein in addition to classical cadherins. To study the role of desmoglein 2 in melanoma cells, melanoma lines containing high endogenous amounts of desmoglein 2 were depleted of the protein by RNA interference. Transwell migration and scratch wounding assays showed markedly increased migration upon desmoglein 2 suppression whereas proliferation and viability remained unaltered. In gene expression profiles, desmoglein 2 depletion was associated with overexpression of migration-related genes. Strongest overexpression was found for secretogranin II which has not been reported in melanoma cells before. The bioactive peptide derived from secretogranin II, secretoneurin, is known to exert chemoattractive functions and was demonstrated here to stimulate melanoma cell migration. In summary, we show that desmoglein 2 expression attenuates migration of melanoma cells. The mechanism of desmoglein 2 impaired cell migration is mediated by downregulation of secretogranin II. Loss of desmoglein 2 increases expression of secretogranin II, followed by an enhanced migratory activity of melanoma cells. Our data add a new pathway of regulating melanoma cell migration related to a desmoglein 2 – secretogranin II axis.

## Introduction

Malignant melanomas are among the most aggressive skin cancers with drastically rising incidence. During their tumorigenesis an important role is attributed to alterations in cell adhesion proteins, in particular, cadherins, calcium-dependent transmembrane glycoproteins mediating homotypic and heterotypic cell-cell interactions [1], [2]. Members of the cadherin superfamily include classical cadherins, that are components of adherens junctions, desmosomal cadherins (desmogleins 1-4 and desmocollins 1-3; [3]), protocadherins and atypical cadherins.

In healthy epidermis, heterotypic adhesions between melanocytes and keratinocytes are mediated by E-cadherin and P-cadherin [4], [5]. However, during melanomagenesis E-cadherin may be downregulated and replaced by N-cadherin [6]. This “cadherin switch” is important for the pathogenesis of melanomas and diverse carcinomas [1], [2], [7]. It provides the melanoma cells with a new adhesive repertoire that enables interactions with new mesenchymal neighbour cells such as fibroblasts [8] and endothelial cells, facilitating invasion and transendothelial migration [8], [9]. N-cadherin promotes proliferation, survival and migration of melanoma cells [9]–[13] whereas E-cadherin counteracts malignancy [14]–[16]. In addition, several other cadherins have been implicated in melanomagenesis. For example, VE-cadherin is associated with a highly aggressive subtype of melanomas and a process designated vascular mimicry [17] whereas P-cadherin [18], H-cadherin [19] and T-cadherin [20] exert tumor suppressive functions.

We have previously shown that certain melanoma cell lines express, in addition to classical cadherins, the desmosomal cadherin desmoglein 2 (Dsg2) [21]. Dsg2 is a widespread transmembrane component of desmosomes in proliferative epithelial cells [3], [22] and a major constituent of the *area composita* of cardiomyocytes, a mixed type of junction [23], [24]. However, in melanoma cells Dsg2 is neither assembled into any cell junction nor found in junctional protein complexes except for plakoglobin but dispersed diffusely over the cell surface [21]. The aim of this study was to analyze the impact of Dsg2 on tumorigenic properties of melanoma cells. We show that depletion of Dsg2 leads to markedly enhanced cell migration, associated with increased expression of migration-related genes, in particular, with upregulation of secretogranin II (SgII) and its chemoattractive peptide secretoneurin (SN).

## Materials and Methods

### Ethics statement

Experiments were conducted with patients’ informed consent and according to the principles of the Declaration of Helsinki and were approved by the Medical Ethics Committee of the Medical Faculty Mannheim, Heidelberg University.

### Antibodies

Murine monoclonal antibodies (mabs) against N-cadherin (clone 32), E-cadherin (clone 36) and β-catenin (clone 14) were purchased from BD Biosciences Pharmingen (Heidelberg, Germany), mabs against Dsg2 (clone 6D8) from Invitrogen (Karlsruhe, Germany), mabs against Dsg1 and 2 (clone DG3.10), plakoglobin (clone 11E4) and polyclonal rabbit antibodies against Dsg2 (clone rb5) from Progen Biotechnik (Heidelberg, Germany). Polyclonal rabbit antibodies against SgII were from GeneTex (GTX116446, affinity-purified antiserum directed against a recombinant fragment within amino acids (aa) 1 and 227 of SgII) and from LifeSpan Biosciences (LS-C39034; raised against the N-terminal sequence (aa 1-19) of rat and human SgII; both obtained through Biozol Diagnostica (Echingen, Germany)). Rabbit antibodies against glyceraldehyde 3-phosphate dehydrogenase (GADPH) from Santa Cruz Biotechnology (Heidelberg, Germany) were used as loading controls for immunoblots. For immunofluorescence microscopy, primary antibody complexes were visualized with secondary antibodies coupled to Cy3 (Dianova, Hamburg, Germany). For immunohistochemistry, we used secondary goat anti-rabbit IgG coupled to horseradish peroxidase (HRP; Santa Cruz Biotechnology). For immunoblot analysis, HRP-conjugated secondary antibodies were applied in combination with the enhanced chemiluminescence system (ThermoFisher Scientific, Schwerte, Germany).

### Cell culture, siRNA transfection and immunoblot

Human melanoma cells of lines MeWo and C32 were obtained from American Type Culture Collection (Manassas, VA, USA). These cell lines were previously characterized in detail with respect to their repertoire of cell adhesion proteins and were found to contain high endogenous amounts of Dsg2 [21]. Cells were cultured in Dulbeccośs Minimal Essential Medium (DMEM) + GlutaMAX TM-L + 4,5 g/L Glucose (Gibco, Invitrogen, Karlsruhe, Germany) supplemented with 10% fetal calf serum (FCS; Biochrom, Berlin, Germany).

Si GENOME smartpool siRNA against human Dsg2 (accession no. NM_001943) was purchased from Dharmacon (obtained through ThermoFisher Scientific). Si GENOME non-targeting siRNA Pool #2 from the same provider was used for control. Transfection was carried out with Dharmafect 1 transfection reagent (Dharmacon, ThermoFisher Scientific) according to the manufacturer’s recommendations. Dsg2 depletion was found to be most efficient after two subsequent siRNA transfections. Cells were split and counted to achieve 30% confluency after 24 h, the time point of the first transfection. One or two days thereafter, cells were subcultured and transfected for the second time with the same siRNA. For analyses of subconfluent cultures, cells were harvested three days after the first and one day after the second transfection. For experiments on confluent cultures they were harvested six days after the first and three or four days after the second transfection.

Immunoblot analysis was performed to compare proteins amounts in Dsg2-depleted cells and controls as described [25], using total protein lysates. Equal amounts of proteins were loaded on each lane. Intensity of bands was quantified with LabImage 1D software (Kapelan Bio-Imaging, Leipzig, Germany).

### Immunofluorescence microscopy and immunohistochemistry

Immunofluorescence microscopy of cultured cells was performed as previously reported [21]. Briefly, cells grown on glass coverslips were fixed in 2% formaldehyde for 5 min, treated with NH_4_Cl for blocking of free aldehyde groups, washed in phosphate-buffered saline (PBS) and permeabilized with 0.1% Triton-X (2–3 min). Primary antibodies were applied for 1 hour (h), secondary antibody for 30 min at room temperature (RT). After a short rinse in distilled water, dehydration in 100% ethanol and air drying, specimens were mounted with Fluoromount-G (Southern Biotech, Biozol Diagnostica). Immunofluorescence microscopic images were captured with an Axiophot II photomicroscope (Carl Zeiss, Jena, Germany) equipped with an AxioCam HR (Carl Zeiss). Confocal laser scanning microscopy was performed with a Zeiss LSM 510 UV microscope.

Immunohistochemistry was conducted on paraffin-embedded primary human melanomas (n = 7), human melanoma metastases (n = 8) and, for control, on Merkel cell carcinomas and normal human skin. Paraffin sections of 1 µm thickness were prepared with a Leica RM2065 microtome (Leica Biosystems, Nussloch, Germany). Sections were deparaffinized in xylol (3×5 min), followed by an ethanol series (100% 2×3 min, 90%, 80% and 70% 1×3 min each), a brief rinse in distilled water and incubation in PBS. Heat-induced antigen retrieval was performed by boiling in Tris-EDTA buffer (pH 9.0; Zytomed Systems, Berlin, Germany) at 100°C for 60 min. After cooling down and washing in PBS (3×5 min) and PBS + 0.1% Tween 20 (30 sec), sections were treated with Dako Peroxidase Blocking Reagent (Dako, Jena, Germany) for 10 min at RT and washed again. Primary rabbit antibodies against SgII (GTX116446 and LS-C39034) and Dsg2 (rb5) were diluted 1:50 in PBS + 1% bovine serum albumin (BSA) and incubated for 60 min at RT, followed by washes in PBS (3×5 min) and PBS + 0.1% Tween 20 (30 sec). Secondary goat anti-rabbit HRP-IgG (Santa Cruz Biotechnology), diluted 1∶100 in PBS + 1% BSA, were applied for 30 min at RT and rinsed off as described above. Sections were developed with AEC Substrate Chromogen (Dako), stained for 1 min with Mayer’s Haemalaun Solution (Merck, Darmstadt, Germany), washed 3×5 min in distilled water and mounted with Dako Faramount Aqueous Mounting Medium. Slides were analyzed and photographed with a Nikon Eclipse microscope equipped with a Nikon Digital Sight DS-Ri1 camera (Nikon, Düsseldorf, Germany). Intensity of the immunoreactions was classified was negative, weakly positive (+), positive (++) or strongly positive (+++). Percentages of immunoreactive cells within one tumor were determined in 10 optical fields at 100-fold magnification.

### Migration and invasion assays

Dsg2-depleted cells and controls were seeded on Transwell migration chambers with 8 µm pore polycarbonate membrane inserts (Corning, Sigma-Aldrich, Deisenhofen, Germany), containing medium with 5% FCS in the upper and 20% FCS in the lower chamber, and allowed to transmigrate for 24 or 48 h. Cells that had not transmigrated were removed with a cotton tip and filters were stained with cell staining solution. The number of transmigrated cells was counted in eight optical fields at 100-fold magnification. For some experiments cells were treated with 10 µg/ml mitomycin C (Carl Roth, Karlsruhe, Germany) for 2 h prior to seeding on the chambers, in order to prevent proliferation. Transwell migration of mitomycin C-treated cells was assessed after 24, 48 and 96 h.

Invasion assays were performed with Matrigel Invasion chambers (BD Biosciences Pharmingen), using FCS gradients between 5 and 20%. The number of invaded cells was determined after 24, 48 and 72 h for MeWo cells and after 48, 72 and 96 h for C32 cells, as described above.

For scratch wounding experiments, confluent monolayers of Dsg2-depleted cells and controls were “wounded” by scratching with a 27-gauze needle. The width of the wound cleft was assessed every two h in ten optical fields, with ten measurements per field and time point. Measurements of each time point were averaged and differences tested for significance. For some experiments, cells were pretreated with 10 µg/ml mitomycin C for 2 h prior to scratching.

### Transepithelial electrical resistance measurement

Our cell-based transepithelial electrical resistance (TEER) assay was applied as previously described [26]–[29]. An epithelial MDCK-C7-cell monolayer was used as a test barrier for malignant cells which will disturb monolayer integrity, followed by decrease and breakdown of TEER. This monolayer develops a high TEER that can be measured continuously using STX-2 electrode (WPI, Sarasota, FL, USA). 10^6^ MDCK-C7 cells were seeded on the reverse side of an upside down oriented membrane filter cup and grown on the filter membrane (growth area: 4.2 cm^2^; pore diameter: 0.4 µm; thickness: 20 µm; Falcon, Heidelberg, Germany). After the MDCK monolayer had reached a high resistance of 15 kΩ/cm^2^, melanoma cells were added into the upper compartment of the filter cup, separated from MDCK-C7 cells by the 20 µm thick filter membrane with 0.4 µm pores and therefore impermeable to both cell types. TEER decrease upon addition of melanoma cells was assessed over a period of 72 h, comparing Dsg2-depleted MeWo and C32 cells to their non-targeting siRNA-treated and untreated counterparts. In control experiments, medium without melanoma cells was added to the MDCK-C7 monolayer. Background electrical resistance built up by filter and medium was constant and extremely low (25 Ω/cm^2^). The maximum TEER of melanoma cells was 30 Ω/cm^2^, a value close to the background resistance. All experiments were performed in duplicate, and measured TEER values were corrected for background resistance.

### Proliferation assay with BrdU incorporation

Proliferation of Dsg2-depleted, non-targeting siRNA-treated and untreated cells was assessed with colorimetric BrdU Cell Proliferation ELISA (Roche Diagnostics, Mannheim, Germany) according to the manufacturer’s recommendations. Subconfluent cell cultures growing for 48 h were incubated with BrdU labelling solution for 24 h. Absorption was measured in an Infinite M200 ELISA reader (Tecan, Crailsheim, Germany) at 400 nm.

### Assessment of cell viability

For viability assays, 10000 and 15000 cells were seeded into 96-well plates in triplicates. Mitochondrial activity of Dsg2-depleted, non-targeting siRNA treated and untreated control cells was quantified by the 3-(4,5-dimethylthiazol-2-yl)-2,5-diphenyltetrazolium bromide (MTT) reduction assay. Viability was measured at 490 nm and expressed as relative values compared to untreated control cells at a density of 10000 cells/well. Treatment with 0.1% Triton X-100 was used as control.

### RNA isolation, cDNA synthesis and real time Reverse Transcriptase PCR

Isolation of total RNA from cultured cells was performed with RNeasy Mini Kit (Quiagen, Hilden, Germany), according to the manufacturer’s instructions. Final RNA concentrations were determined with an Ultrospec 3100pro photometer (Amersham Biosciences, Freiburg, Germany), and quality was checked on formaldehyde-containing 1% agarose gels. For long-term storage at –80°C, RNA was precipitated with 2.5 volumes ethanol and 0.1 volume 3 M sodium acetate (pH 5.2).

Synthesis of cDNA was conducted with 1 µg total RNA as template. After incubation with 1 µl Oligo (dt)18 primer and RNAse free water at 65°C for 10 min, samples were placed on ice. M-MuLV RT buffer, Ribo Lock RNAse Inhibitor, 0.5 mM dNTPs and RevertAid H Minus M-MuL V Reverse Transcriptase (all from Fermentas, St. Leon-Rot, Germany) were added, and samples were incubated first for 60 min at 42°C, then for 10 min at 70°C.

For real time RT-PCR 1.75 µl of cDNA was amplified using SYBR Green PCR Master Mix (Applied Biosystems, Life Technologies GmbH, Darmstadt, Germany) under standard conditions with a Stratagene MX3005P sequence detection system (Agilent Technologies, Böblingen, Germany). All experiments were repeated at least in triplicates and expression was normalized to β-actin expression. The following primers for human Dsg2, β-actin and SgII were used: Dsg2 forward TGG ACA CCC AAA CAG TGG CCC T; Dsg2 reverse CTC ACT TTG TTG CAG CAG CAC AC; actin forward GGC ACC ACA CCT TCT ACA ATG A; actin reverse TCT CCT TAA TGT CAC GCA CGA T; SgII-1 forward CCA GGT CAC TGG GGA GTC TGC T; SgII-1 reverse TGA GCA TCA ACA ATG CCA; SgII-2 forward TCC CAC CCC AAG CAA ATC CCA AC; SgII-2 reverse TGA AGC AGA CTC CCC AGT GAC CTG.

### Gene expression profiling

Gene expression profiling was performed by Dr. M. Scharfenberger-Schmeer (Department of Genomics and Proteomics, German Cancer Research Center, Heidelberg), using Illumina human Sentrix-12 microarrays. Triplicates of RNA samples were gained from subconfluent cultures of Dsg2-depleted and non-targeting siRNA-treated MeWo and C32 cells. The quality of total RNA was checked by gel analysis using the total RNA Nano chip assay on an Agilent 2100 Bioanalyzer (Agilent Technologies GmbH, Berlin, Germany). Only samples with RNA index values greater than 8.5 were selected for expression profiling. RNA concentrations were determined using the NanoDrop spectrophotometer (NanoDrop Technologies, Wilmington, DE, USA).

Biotin-labeled cRNA samples for hybridization on Illumina human Sentrix-12 BeadChip arrays (Illumina, Inc., Amplifa Labortechnik GmbH, Wasserburg Bodensee, Germany) were prepared according to Illumina's recommended sample labeling procedure based on the modified Eberwine protocol [30]. In brief, 500 ng total RNA was used for cDNA synthesis, followed by an amplification/labeling step *(in vitro* transcription) to synthesize biotin-labeled cRNA according to the MessageAmp II aRNA amplification kit (Ambion, Inc., Austin, TX, USA). Biotin-16-UTP was purchased from Roche Applied Science (Penzberg, Germany). The cRNA was column purified according to TotalPrep RNA Amplification Kit and eluted in water. Quality of cRNA was checked using the RNA Nano Chip Assay on an Agilent 2100 Bioanalyzer and spectrophotometrically quantified (NanoDrop Technologies).

Hybridization was performed at 58°C in GEX-HCB buffer (Illumina Inc.) at a concentration of 100 ng cRNA/µl, unsealed in a wet chamber for 20 h. Spike-in controls for low, medium and highly abundant RNAs as well as mismatch control and biotinylation control oligonucleotides were added. Microarrays were washed once in High Temp Wash buffer (Illumina Inc.) at 55°C and then twice in E1BC buffer (Illumina Inc.) at room temperature for 5 minutes. After blocking for 5 min in 4 ml of 1% (wt/vol) Blocker Casein in PBS Hammarsten grade (Pierce Biotechnology, Inc., Rockford, IL, USA), array signals were developed by a 10-min incubation in 2 ml of 1 µg/ml Cy3-streptavidin (Amersham Biosciences, Buckinghamshire, UK) solution and 1% blocking solution. After a final wash in E1BC, arrays were dried and scanned.

Microarray scanning was done using a Beadstation array scanner, setting adjusted to a scaling factor of 1 and PMT settings at 430. Data were extracted for all beads individually, and outliers were removed at >2.5 median absolute deviation. All remaining data points were used for the calculation of the mean average signal for a given probe, and standard deviation (SD) for each probe was calculated. Data analysis was performed by normalization of the signals using the quantile normalization algorithm without background subtraction. Differentially regulated genes were defined by calculating the SD differences of a given probe in a one-by-one comparison of samples or groups.

### Radioimmunoassay

Concentrations of intracellular SN in Dsg2-depleted versus (vs.) non-targeting siRNA-treated MeWo and C32 cells were determined by a specific radioimmunoassay (RIA). Cell extracts were obtained from subconfluent cultures of twice sequentially transfected cells. After three washes with PBS, cells were scraped off the culture dish and centrifuged for 5 min at 2000 g. Pellets were resuspended in 500 µl distilled water, sonicated, boiled for 10 min and centrifuged for 20 min at 10000 g. Supernatants were lyophilized and analyzed for SN by RIA as described by Kirchmair et al. [31]. The detection limit of the RIA was 1 fmol.

### Live cell microscopy

Live cell microscopy was performed to compare migratory capacities of SN-stimulated vs. unstimulated C32 cells, using a Zeiss Axio Observer Z.1 microscope equipped with AxioVision 4.8 software (Carl Zeiss AG, Oberkochen, Germany). C32 cells were seeded on a glass coverslip coated precoated with 50 µg/ml fibronectin (Roche Diagnostics) and allowed to adhere for 3 h in HEPES buffer. 10^−6^ M human SN (PolyPeptide Laboratories, Strasbourg, France) was added immediately before recording was started. Images were captured at 120-sec intervals at 100-fold magnification. The distance covered by each cell within 3.5 h was determined by Time Lapse Analyzer v01_32 software (AG Bioinformatics and Systems Biology, Institute of Neural Information Processing, Institute of Virology, University of Ulm, Germany).

### Statistical analysis

Each experiment was performed at least in triplicates unless stated otherwise. Differences between Dsg2-depleted cells and controls were tested for statistical significance with pairwise t-tests, using Prism 5 GraphPad software. A p-value ≤0.05 was considered significant.

## Results

### Knockdown of Dsg2 in cultured melanoma cells

**Figure 1:**
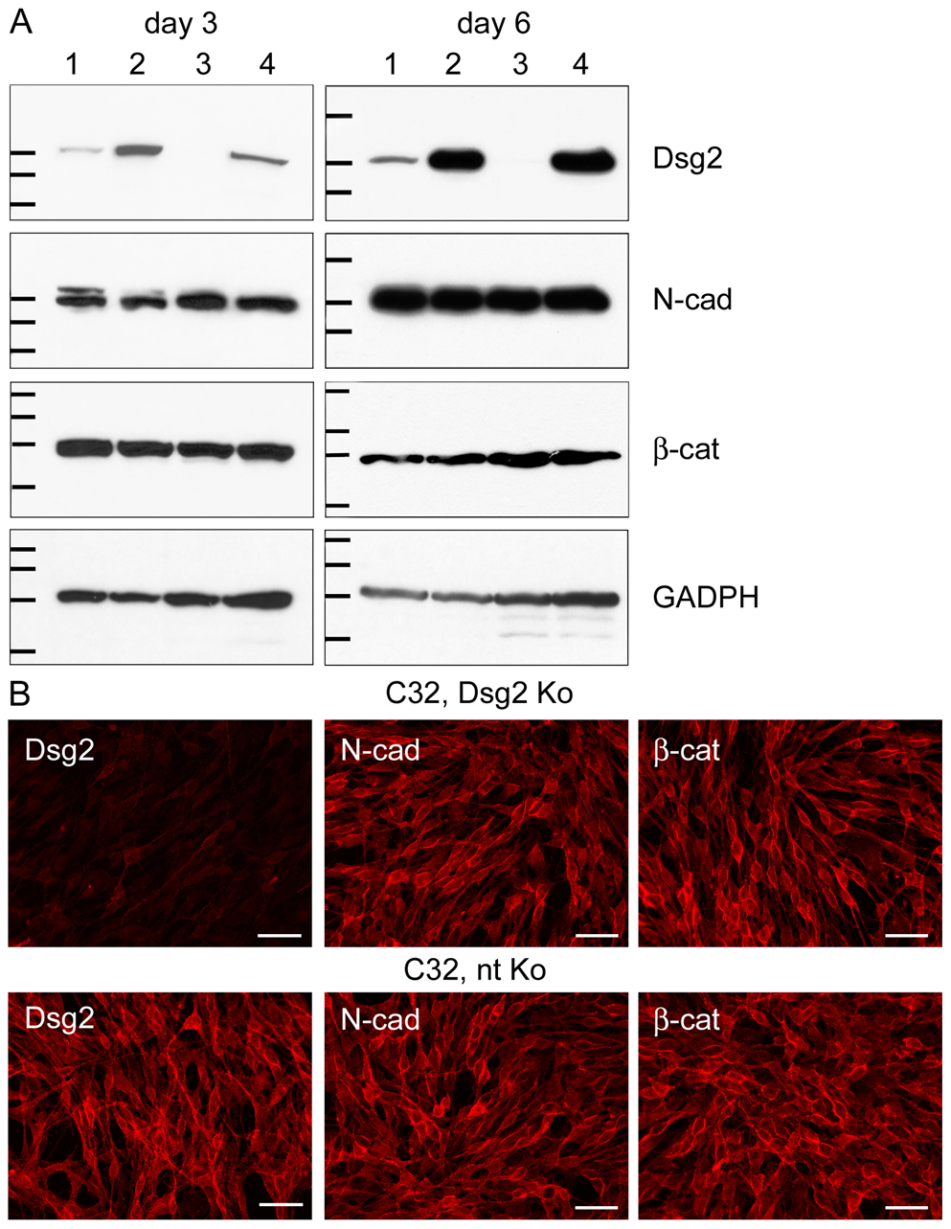
No alterations in N-cadherin and β-catenin after knockdown of Dsg2 in melanoma cells. (A) Immunoblots showing efficient Dsg2 depletion in MeWo and C32 melanoma cells. Equal amounts of proteins were loaded. 1: MeWo, Dsg2 siRNA; 2: MeWo, non-targeting (nt) siRNA; 3: C32, Dsg2 siRNA; 4: C32, nt siRNA. In MeWo, Dsg2 reduction was 7.9-fold three days after Dsg2 siRNA transfection and 5.1-fold six days thereafter when the intensity of the bands was normalized against the GADPH immunoblots serving as loading controls. In C32, Dsg2 was 12.7-fold or 122.8-fold reduced. By contrast, protein amounts of N-cadherin (N-cad) and β-catenin (β-cat) were virtually unchanged upon Dsg2 depletion. Molecular weight markers (from top to bottom): Dsg2 immunoblots: 158, 116 and 97.2 kDa (day 3); 212, 158 and 116 kDa (day 6); N-cad immunoblots: 116, 97.2 and 66.4 kDa (day 3); 158, 116 and 97.2 kDa (day 6); β-cat immunoblots: 158, 116, 97.2 and 66.4 kDa (day 3 and 6); GADPH immunoblots: 55.6, 42.7, 34.6 and 27 kDa (day 3 and 6). (B) Immunofluoresence microscopy of Dsg2-depleted (upper panel) and nt siRNA-treated C32 cells (lower panel), showing virtual absence of Dsg2 three days after knockdown. In cells treated with nt siRNA Dsg2 is accumulated at the cell surface and at cell borders. Antibodies to N-cad and β-cat react at cell-cell junctions and along cell borders, in patterns unaffected by Dsg2 contents. Bars: 200 µm.

Melanoma cell lines previously demonstrated to contain high endogenous amounts of Dsg2, i.e., MeWo cells derived from a lymph node metastasis and C32 cells from a primary amelanotic melanoma [21], were depleted of Dsg2 using siRNA techniques and compared to their Dsg2-rich counterparts treated with non-targeting siRNA. As knockdown was optimal after two sequential siRNA treatments, this approach was used for all subsequent experiments. Successful depletion of Dsg2 in subconfluent and confluent cultures (“day 3” and “day 6”) was confirmed by immunoblot (Fig. 1A). Protein contents of N-cadherin and β-catenin were unchanged upon Dsg2 depletion (Fig. 1A). Moreover, the subcellular localization of these and other adhering junction-associated proteins was unaltered. Representative micrographs of Dsg2-depleted and non-targeting siRNA-treated C32 cells immunostained with antibodies against Dsg2, N-cadherin and β-catenin are shown in Fig. 1B. E-cadherin was neither detected in untreated MeWo or C32 cells, corresponding to our previous observations [21], nor in MeWo or C32 cells treated with Dsg2 siRNA or non-targeting siRNA. Furthermore, MeWo and C32 cells did not contain any other desmosomal proteins, except trace amounts of plakoglobin in MeWo that remained barely detectable by Western Blot and immunostaining after depletion of Dsg2 (see also [21]).

### Dsg 2 knockdown leads to increased cell migration

**Figure 2:**
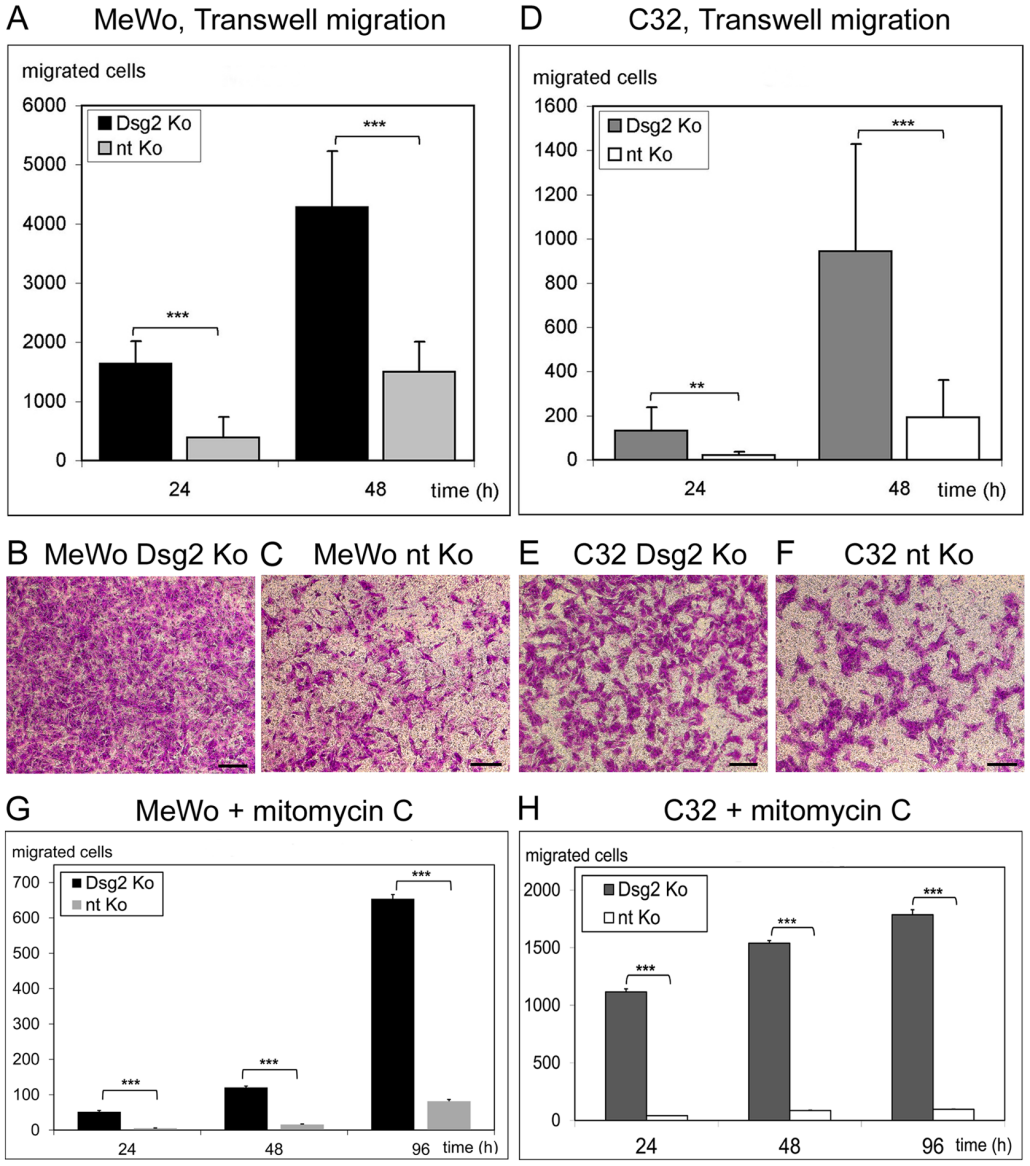
Depletion of Dsg2 leads to enhanced migration of melanoma cells. (A-F) Transwell migration assays comparing Dsg2-depleted (“Dsg2 Ko”) and non-targeting siRNA-treated (“nt Ko”) MeWo and C32 cells 24 and 48 h after seeding. (A, D) At both time points and in both cell lines substantially more Dsg2-depleted cells than controls had migrated through the filter. (B, C, E, F) Micrographs of hematoxylin-eosin stained filter bottoms taken after 48 h. Bars: 300 µm. (G, H) Transwell migration assays of Dsg2-depleted and non-targeting siRNA-treated MeWo (G) and C32 (H) preincubated with mitomycin C. Bars: SD; * p≤0.05, ** p≤0.01, *** p≤0.001.

In Transwell migration assays depletion of Dsg2 was associated with markedly increased migration (Fig. 2). The ratio of transmigrated Dsg2-depleted MeWo cells to transmigrated MeWo controls was 4.2 after 24 h (p<0.001) and 2.9 after 48 h (p<0.001; Fig. 2A). For C32 cells accordant ratios were 6.1 after 24 h (p = 0.0066) and 4.9 after 48 h (p<0.001; Fig. 2D; for examples of hematoxylin-eosin stained filter bottoms, see Fig. 2B, C, E, F). When MeWo and C32 cells were pretreated with mitomycin C to abolish proliferation, differences in transmigration between Dsg2-depleted cells and controls were even more pronounced (Fig. 2G, H). 9.8-fold more Dsg2-depleted MeWo than controls had transmigrated after 24 h (p<0.001), 7.5-fold more after 48 h (p<0.001), and 8.0-fold more after 96 h (p<0.001; Fig. 2G). In the C32 cell line, ratios between transmigrated Dsg2-depleted cells and transmigrated controls were 27.9 (p<0.001), 18.2 (p<0.001) and 18.6 (p<0.001) after 24, 48 and 96 h (Fig. 2H).

**Figure 3:**
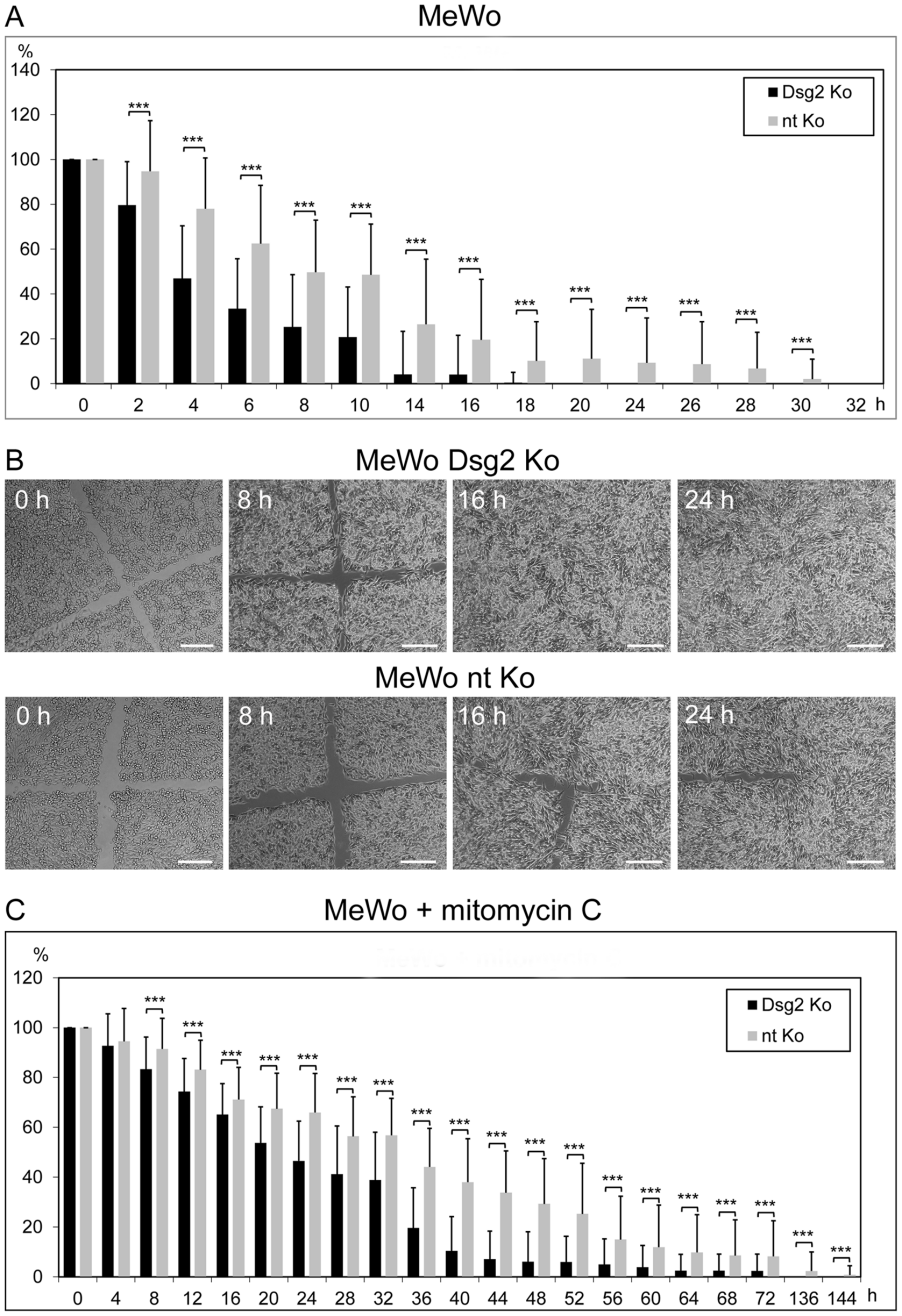
Scratch wounding assays demonstrating accelerated wound closure after Dsg2 depletion. Confluent monolayers of Dsg2-depleted (“Dsg2 Ko”) or non-targeting siRNA-treated (“nt Ko”) MeWo were wounded by scratching. (A) Bar diagram showing the percental width of the wound cleft at different time points after scratching. (B) Representative micrographs of wound clefts taken 0, 8, 16 and 24 h after scratching. Bars: 200 µm. (C) Scratch wounding experiment of Dsg2-depleted and non-targeting siRNA-treated MeWo cells that had been preincubated with mitomycin C to abolish proliferation. Bars: SD; * p≤0.05, ** p≤0.01, *** p≤0.001.

Correspondingly, scratch wounding assays of Dsg2-treated MeWo cells and controls treated with non-targeting siRNA demonstrated significantly faster wound closure after Dsg2 depletion (Fig. 3A; time until complete wound closure: 18 vs. 32 h; for examples of micrographs of the wound clefs taken at different time points after scratching see Fig. 3B). The same observation was made when cells were pretreated with mitomycin C prior to scratch wounding (Fig. 3C). C32 cells were not systematically assessed in scratch wounding assays, since in preliminary scratch experiments they turned out to migrate considerably more slowly than MeWo cells.

### Impact of Dsg2 on cell invasion

**Figure 4:**
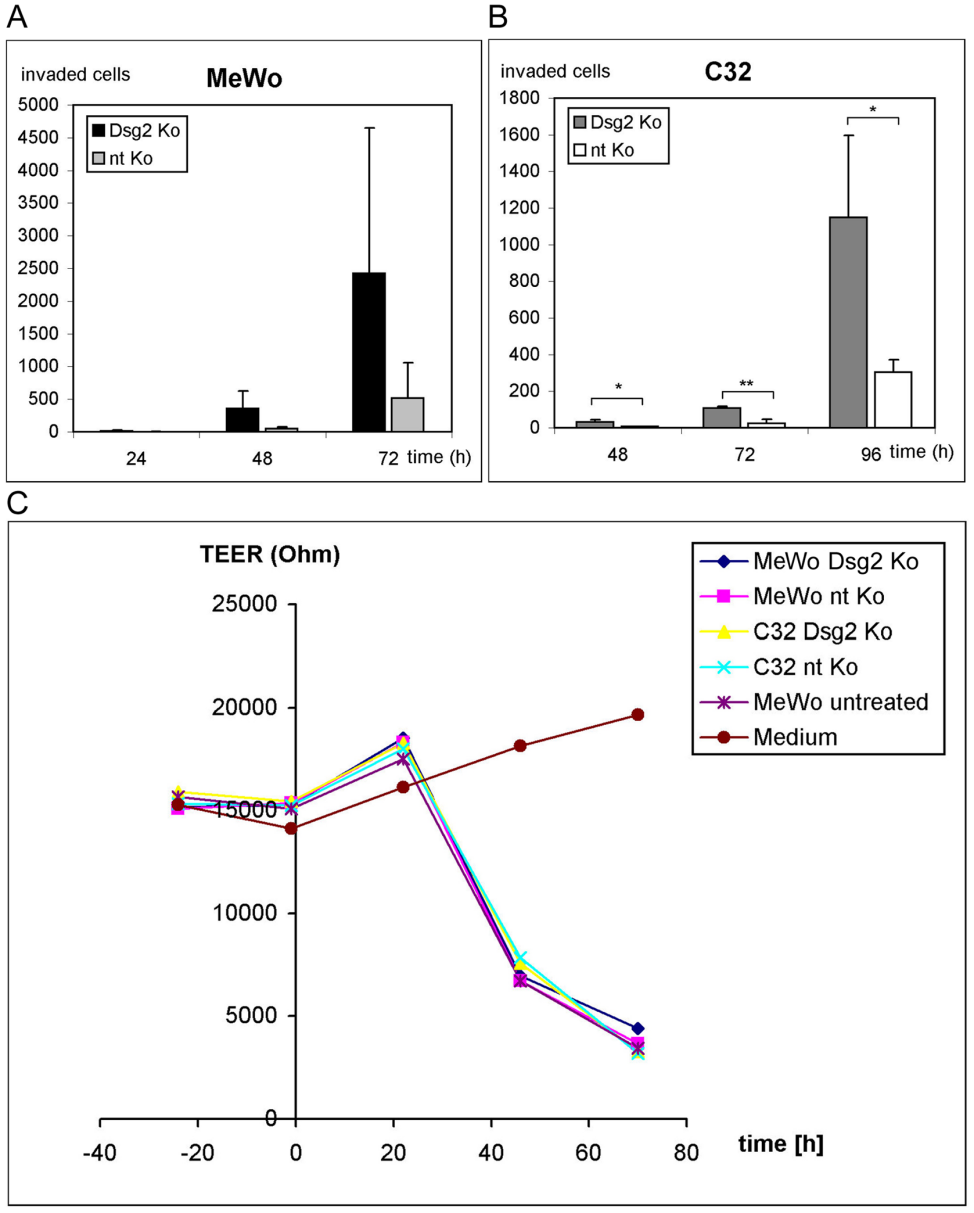
Impact of Dsg2 on invasion. (A, B) Transwell invasion assays of Dsg2-depleted (“Dsg2 Ko”) or non-targeting siRNA-treated (“nt Ko”) MeWo and C32. Considerably more Dsg2 Ko than nt Ko MeWo had invaded after 24, 48 and 72 h (A); however, differences did not achieve significance. (B) In the line C32, the number of invaded cells was significantly increased upon Dsg2 knockdown after 48, 72 and 96 h. Bars: SD; * p≤0.05, ** p≤0.01. (C) TEER assay, showing no alteration of invasive properties upon Dsg2 depletion. A marked decrease in TEER was seen in cocultures of Dsg2 Ko, nt Ko or untreated MeWo and C32 with a MDCK-C7 monolayer after 48 and 72 h. However, no significant differences were noted between Dsg2-depleted and control cells.

Transwell invasion assays showed appreciably increased invasion of melanoma cells upon Dsg2 depletion. Ratios of Dsg2-depleted vs. non-targeting siRNA-treated MeWo migrated through a Matrigel-coated Transwell filter were 5.6, 7.3 and 4.7 after 24, 48 and 72 h. However, measured differences did not reach statistical significance, due to large variations (Fig. 4A). Significantly more Dsg2-depleted C32 than C32 controls had invaded and transmigrated after 48, 72 and 96 h, with ratios of 5.2, 4.4 and 3.8 (p = 0.014, p = 0.004 and p = 0.032; Fig. 4B).

Since the Transwell invasion assay does not reflect interactions between cancer cells and normal tissue, a cell-based TEER breakdown assay was used to test invasion under more physiological conditions [27], [28]. Dsg2-depleted MeWo and C32 and control cells were cocultured with a confluent monolayer of epithelial MDCK-C7 cells with high TEER (15 kΩ/cm^2^). To test the impact of Dsg2 on invasive properties unrelated to migration, melanoma cells and MDCK-C7 were separated by a filter membrane anticipating physical contact. TEER decreased to 7.5 kΩ/cm^2^ after 48 h of coculture and breakdown proceeded to <5 kΩ/cm^2^ after 72 h (Fig. 4C). However, no differences were observed between Dsg2-depleted cells and controls (Fig. 4C). These data show that Dsg2 depletion increases the migratory activity but keeps the invasive properties of melanoma cells constant.

### Dsg2 does not influence proliferation and viability of melanoma cells

**Figure 5:**
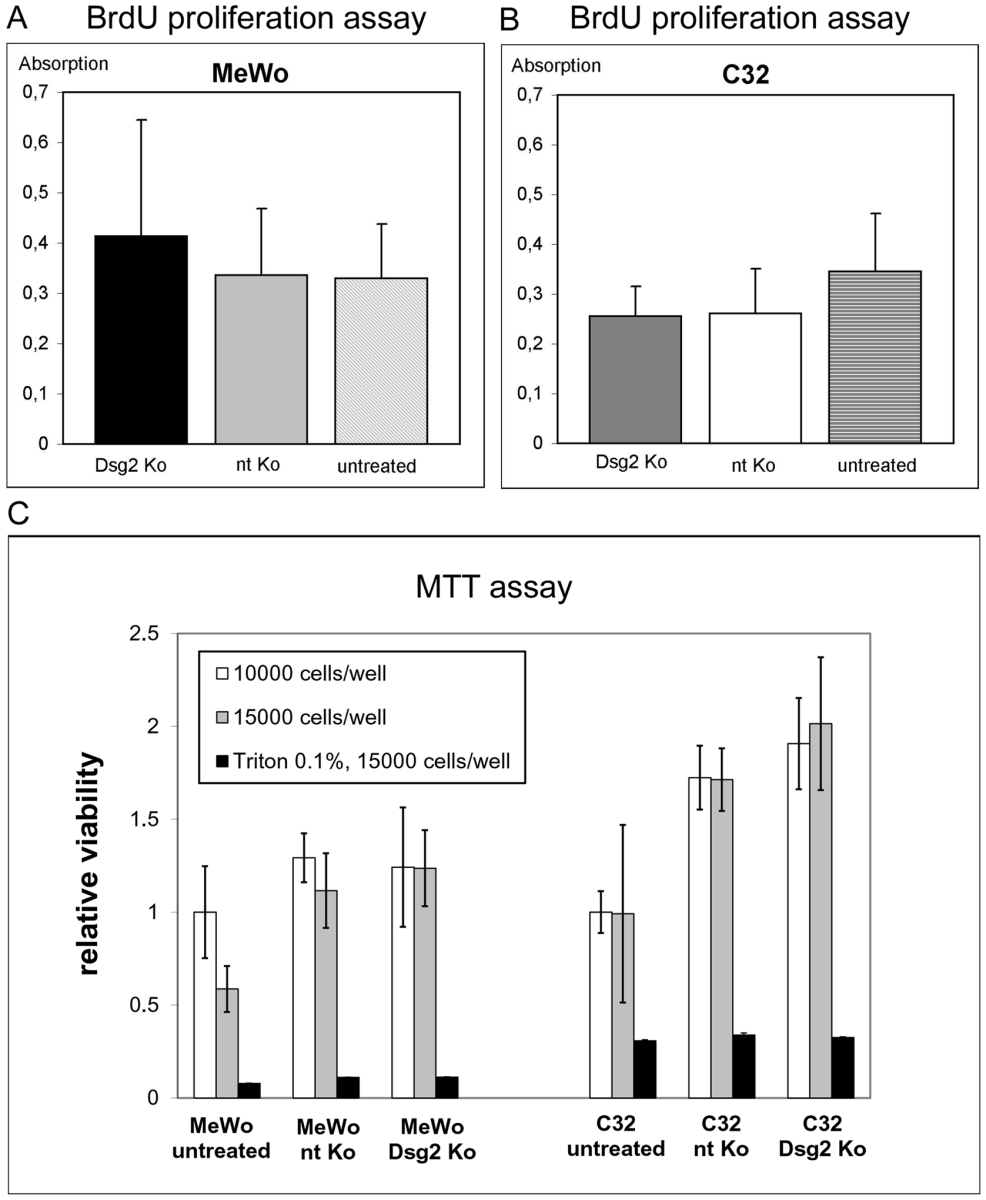
Dsg2 depletion does not significantly influence proliferation and viability of melanoma cells. (A, B) Bar diagrams showing the absorption rates determined with colorimetric BrdU Cell Proliferation ELISA. Dsg2-depleted MeWo (“Dsg2 Ko”; A) and Dsg2-depleted C32 cells (B) were compared to cells treated with non-targeting siRNA (“nt Ko”) and to untreated cells. Pairwise t-tests gave no significant differences with respect to absorption, indicating similar proliferation rates in all probes. (C) MTT assay comparing the viability of Dsg2-depleted MeWo and C32 cells to non-targeting siRNA-treated and untreated controls at densities of 10000 cells/well (white bars) or 15000 cells/well (grey bars). Quantification and normalization showed no significant differences between Dsg2 Ko and nt Ko cells. Analysis of untreated cells indicated increase of mitochondrial activity induced by the transfection technique. Viability of Dsg2 Ko and nt Ko MeWo and C32 cells is expressed as relative values compared to untreated cells at a density of 10000 cells/well. Treatment with 0.1% Triton X-100 was used as control. Bars: SD.

BrdU incorporation assays of Dsg2 siRNA-treated, non-targeting siRNA-treated and untreated MeWo and C32 cells showed similar proliferation rates in all samples, measured at the same time after Dsg2 knockdown as indicated for migration and invasion experiments (Fig. 5A, B).

In order to evaluate effects of Dsg2 knockdown on cell viability, mitochondrial activity was assessed using the MTT reduction method (Fig. 5C). Compared to treatment with non-targeting siRNA, depletion of Dsg2 had no effect on the viability of MeWo cells at cell densities of 10000 cells/well or 15000 cells/well. Similarly, MTT assays comparing Dsg2-depleted C32 to their non-targeting siRNA-treated counterparts revealed no significant differences in viability (Fig. 5C). To investigate the impact of the transfection procedure on the results, we additionally analyzed the mitochondrial activity of untreated cells. Quantitative analysis showed that transfection with non-targeting siRNA or with Dsg2 siRNA correlates with an increased mitochondrial activity in MeWo cells at a density of 15000 cells/well. Similar results were obtained for C32 cells demonstrating an approximately twofold increase in metabolic activity after siRNA treatment. However, measurement of LDH release due to membrane damage demonstrated that the transfection of melanoma cells did not induce cytotoxicity (data not shown). Therefore, Dsg2 appears to affect neither proliferation nor viability of melanoma cells.

### Upregulation of migration-related genes upon Dsg2 depletion

To test for differential gene expression upon Dsg2 depletion, RNA extracts of subconfluent Dsg2-depleted and non-targeting siRNA-treated MeWo and C32 were analyzed with Illumina human Sentrix-12 microarrays. Expression profiles were compared with GoMiner software [32] to systematically predict biological processes and pathways correlated with Dsg2 depletion.

In Dsg2-depleted C32 vs. C32 controls, expression of the DSG2 gene was 7.65-fold decreased. In addition, 357 genes were up- or downregulated more than 1.5-fold. Dsg2 knockdown was significantly associated with differential expression of genes involved in cell migration, cell motility, anatomical structure morphogenesis, cell adhesion, developmental processes, protein kinase cascades (in particular, the mitogen-activated protein kinase (MAPK) cascade), transforming growth factor beta (TGF-β) receptor signaling and transmembrane receptor protein serine threonine kinase signaling (Table S1).

**Figure 6:**
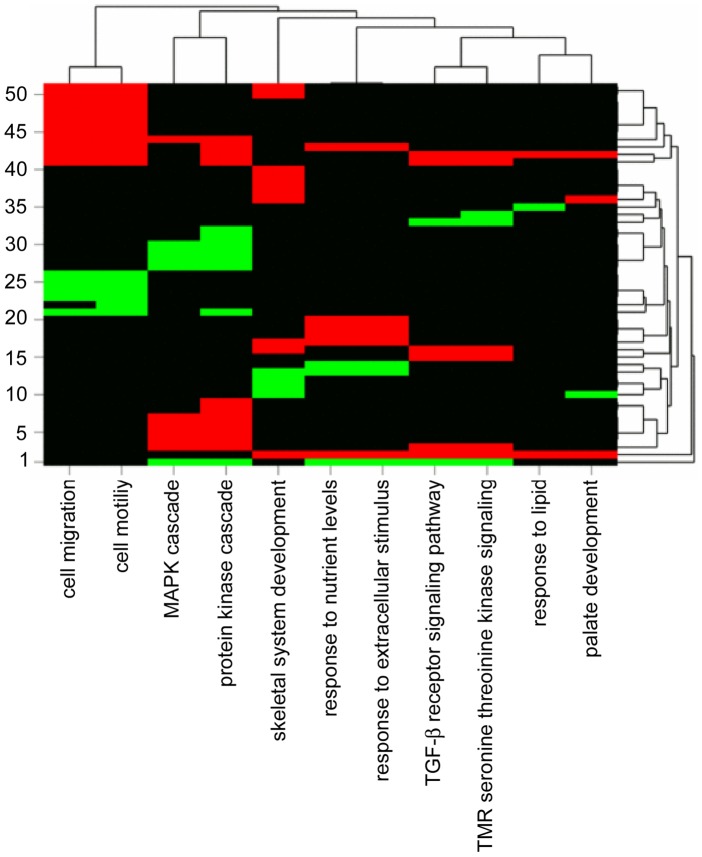
Clustered image map of >1.5-fold up- or downregulated genes in Dsg2-depleted C32. *1. CAV1,* 2. TGFBR2, 3. DUSP22, 4. STRADB, 5. RASGRP3, 6. CBS, 7. MDFIC, 8. GOLT1B, 9. SRPK2, *10. COL11A2, 11. DLX5, 12. SPARC, 13. SPP1, 14. TULP4,* 15. C5ORF13, 16. COL1A2, 17. MGP, 18. KYNU, 19. ASNS, 20. ATG12, *21. SRC, 22. ROPN1B, 23. VCAN, 24. PPAP2B, 25. OPHN1, 26. PLXNB1, 27. TIMP2, 28. RAPGEF2, 29. EFNA1, 30. MYD88, 31. RPS6KA5, 32. ZDHHC17, 33. CITED1, 34. FSTL1, 35. VPS4B,* 36. PRRX1, 37. PHEX, 38. PAPSS1, 39. CDK6, 40. MYC, 41. CCL2 42. TGFBR3, 43. HMOX1, 44. SCG2, 45. LAMC1, 46. LAMA4, 47. IL8, 48. CEACAM1, 49. FN1, 50. CTGF, 51. IGFBP5. Upregulated genes are highlighted in red (bold print) and downregulated genes in green (italics). Clusters are based on euclidean distance.

Separate analysis of up- or downregulated genes demonstrated significant correlations between Dsg2 knockdown and overexpression of genes related to cell migration (3.96-fold enrichment, p<0.0001, false discovery rate (FDR) 0.0267), TGF-β receptor signaling (6.60-fold enrichment, p<0.0001, FDR 0.0413) and cell adhesion (2.68-fold enrichment, p = 0.0002, FDR 0.0300). A cluster image map of differentially expressed genes is shown in Fig. 6.

In Dsg2-depleted MeWo vs. MeWo controls expression of Dsg2 was 5.06-fold decreased and 87 additional genes were >1.5-fold up- or downregulated. Significant enrichment was noted for genes involved in cell motility (2.90-fold, p = 0.0288), protein kinase cascades (3.12-fold, p = 0.0069), MAPK cascade (4.20-fold, p = 0.0151), TGF-β receptor signaling (5.89-fold, p = 0.0143) and transmembrane receptor protein serine threonine kinase signaling (6.34-fold, p = 0.0036). However, FDRs were insignificant.

Combining expression profiles of MeWo and C32 cells, 46 genes were >1.5-fold differentially expressed (Table S2). Strongest upregulation (3.57-fold in C32 and 2.27-fold in MeWo) was found for the secretogranin II gene (SCG2), encoding the motility-related protein SgII. SgII is typically produced by neuronal, endocrine and immune cells, but also induced in other cell types under pathological conditions like hypoxia [33]. Interestingly, this protein has not been reported before in melanoma cells.

### Secretogranin II and secretoneurin are upregulated upon Dsg2 depletion and stimulate melanoma cell migration

**Figure 7:**
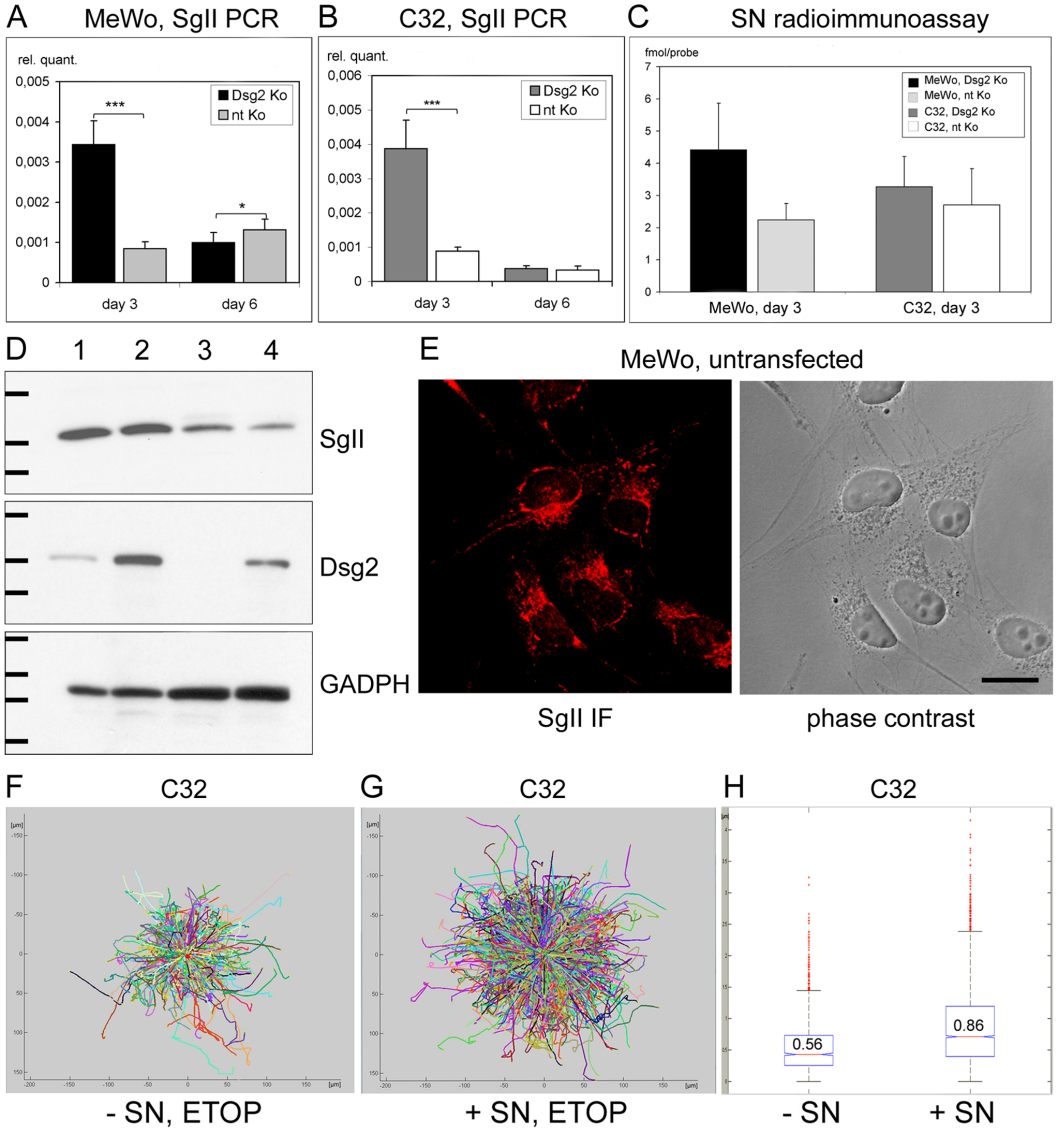
Upregulation of SgII and SN upon Dsg2 depletion and increased melanoma cell migration upon stimulation with SN. (A, B) Real time PCR showing significantly increased SgII mRNA in subconfluent but not in confluent Dsg2-depleted MeWo and C32. In subconfluent cultures harvested three days after the first siRNA transfection, 4.1-fold more SgII mRNA (MeWo, A) or 4.4-fold more SgII mRNA (C32, B) was detected after Dsg2 knockdown. However, in confluent cultures harvested after six days amounts of SgII mRNA were equal in the C32 samples (B) and slightly decreased in Dsg2-depleted MeWo (1.3-fold, A). (C) RIA demonstrating marked upregulation of SN in cellular extracts of Dsg2-depleted MeWo (4.42 vs. 2.24 fmol/probe, p = 0.0704) and slight SN increase in Dsg2-depleted C32. Bars: SD; * p≤0.05, *** p≤0.001. (D) Immunoblots of total cell lysates from subconfluent Dsg2-depleted MeWo (lane 1), nt siRNA-treated MeWo (lane 2), Dsg2-depleted C32 (lane 3) and nt siRNA-treated C32 (lane 4), harvested three days after the first siRNA transfection. SgII (detected with rabbit antiserum GTX116446) was upregulated 1.6-fold in Dsg2-depleted MeWo as compared to MeWo controls and 1.5-fold in Dsg2-deleted C32 as compared to C32 controls when the intensity of the bands was normalized against the GADPH blot serving as loading control. Dsg2 was depleted 3.1-fold (MeWo) or 26.3-fold (C32) after knockdown. Molecular weight markers (from top to bottom): SgII immunoblot: 97.2, 66.4 and 55.6 kDa; Dsg2 immunoblot: 212, 158 and 116 kDa; GADPH immunoblot: 55.6, 42.7, 34.6 and 27 kDa. (E) Confocal laser scanning micrographs of untreated MeWo cells stained with SgII antibodies (GTX116446). IF – immunofluorescence. Bar: 20 µm. (F-H) Enhanced melanoma cell migration after stimulation with SN. Migration of unstimulated (“- SN”) or SN-stimulated (“+ SN”) C32 on fibronectin was documented by live cell microscopy for 3.5 h. (D, E) Equalize track origins plots reveal larger migration distances after SN stimulation. (F) Bar diagram presenting average mean displacement, 25^th^ and 75^th^ percentiles (box) and individual measurements (red dots).

Real time PCR comparing subconfluent Dsg2-depleted MeWo and C32 cells to their non-targeting siRNA-treated counterparts revealed a >4-fold upregulation of SgII mRNA upon Dsg2 knockdown in both lines, well in accordance with the results from gene expression profiling (p<0.001; Fig. 7A, B). By contrast, in confluent cultures amounts of SgII mRNA were either equal (C32; Fig. 7B) or slightly decreased (MeWo; Fig. 7A) after Dsg2 depletion, although Dsg2 mRNA was efficiently reduced in all Dsg2 siRNA-treated samples (i.e., 4.9- or 4.8-fold in subconfluent or confluent MeWo cultures and 18.5- or 10.2-fold in subconfluent or confluent C32 cultures; data not shown). The observation that SgII is upregulated only in subconfluent – i.e., potentially migratory – Dsg2-depleted cells but not in confluent cultures was confirmed with a second pair of SgII-specific primers (data not shown). Moreover, this finding is in accordance with gene expression profiling data from confluent MeWo and C32 cultures that did not show differential expression of the SCG2 gene upon Dsg2 depletion (not shown).

When cell extracts of subconfluent MeWo cultures were analyzed by RIA, the bioactive peptide SN was 1.97-fold upregulated upon Dsg2 depletion (p = 0.0704; Fig. 7C). Cell extracts of Dsg2-depleted C32 cells contained 1.21-fold more SN than controls (not significant, Fig. 7C). Immunoblot analysis revealed 1.6-fold higher amounts of SgII in subconfluent Dsg2-depleted MeWo cells compared to non-targeting siRNA-treated MeWo controls (Fig. 7D). In C32 cells the SgII protein was 1.5-fold increased after Dsg2 knockdown (Fig. 7D). The intensity of the SgII bands was normalized against the GADPH blots serving as controls.

Immunostaining of untransfected MeWo and C32 cells with antibodies against SgII demonstrated SgII-positive immunoreactions in perinuclear and cytoplasmic granular structures. Representative confocal laser scanning micrographs of SgII-labelled MeWo cells are shown in Fig. 7E. When Dsg2-depleted MeWo and C32 cells were compared to untreated and non-targeting siRNA-treated controls, SgII-positive immunoreactions appeared somewhat more intense upon Dsg2 depletion. However, we abstained from quantitative comparisons of the fluorescence intensity, since this method does not allow reliable estimation of protein amounts (data not shown).

In other cell types SN exerts chemoattractive and pro-migratory functions [34]-[37]. Pro-migratory effects of SN on melanoma cells were exemplary demonstrated by live cell microscopy of SN-stimulated vs. unstimulated C32 cells (Fig. 7F-H). Average mean distances covered per minute by single cells on fibronectin were 0.56 µm in unstimulated cells. Stimulation with 10^−6^ M SN increased distances to 0.86 µm (p<0.05; Fig. 7H). Equalize track origins plots, showing distances covered by each individual SN-stimulated or unstimulated cell, are presented in Fig. 7F and G. Taken together, these findings substantiate our hypothesis that Dsg2 knockdown leading to an upregulation of SgII and SN promotes migration of melanoma cells.

**Figure 8:**
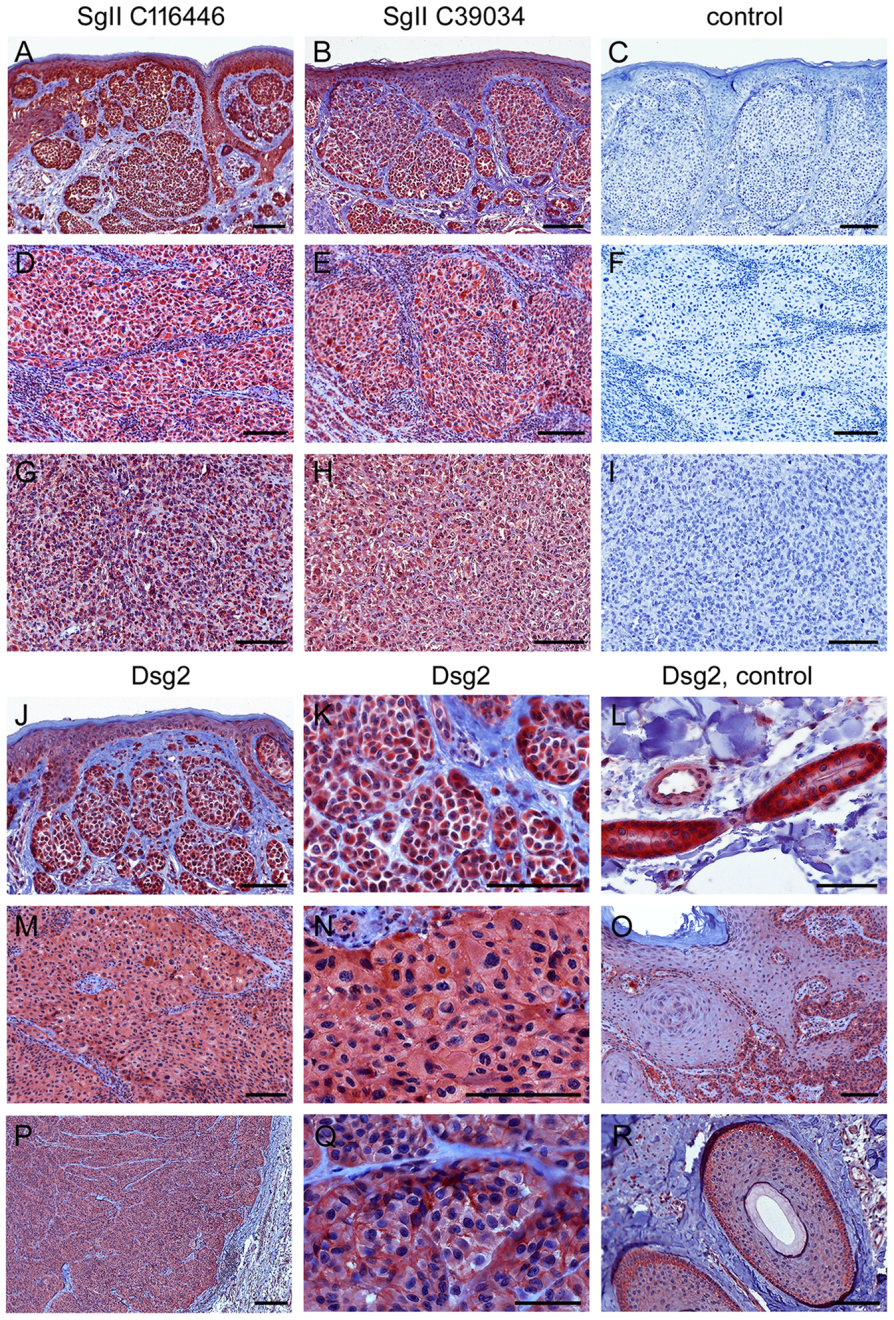
Immunohistochemistry of SgII and Dsg2 on paraffin sections of primary malignant melanomas and melanoma metastases. (A-C, J-L) Primary nodular malignant melanoma (NMM) with a tumor thickness of 0.8 mm (pT1a; no. 4 in the Table S3). (D-F, M-O) Primary NMM of 10 mm thickness (pT4a; no. 1). (G-I) Melanoma metastasis of the mamma (no. 8). (P-R) Cutaneous melanoma metastasis of the temple (no. 15). In the upper part of the figure (A-I), tumors were immunostained with two different SgII antisera (A, D, G: GTX116446 and B, E, H: LS-C39034), or, for control, with secondary goat anti-rabbit HRP-IgG (C, F, I). Clearly positive immunoreactions are seen in the cytoplasm of the melanoma cells with both SgII antisera in all tumors examined. In the lower part (J-R), tumors were reacted with antibodies against Dsg2 (rb5). Diffuse Dsg2-positive reactions are detected in the cytoplasm and/or at the surface of the melanoma cells (J, M, P: overviews; K, N, Q: higher magnifications). In addition, Dsg2 appears to be focally enhanced at the cell-cell contacts of the primary NMM of 10 mm thickness and the cutaneous metastasis (N, Q). Dsg2-positive structures serving as internal positive controls on each slide, i.e. sweat glands (L), basal epidermis (O) and hair follicles (R) are shown on the right hand side of each row. Bars: 50 µm (L, Q), 200 µm (P) or 100 µm (all other micrographs).

To investigate whether malignant melanomas contain SgII *in vivo*, we performed immunohistochemistry on a sample of human primary melanomas (n = 7) and melanoma metastases (n = 8; for details, see Table S3). In all tumors analyzed, clearly SgII-positive reactions were detected in the cytoplasm of the melanoma cells (for examples of primary melanomas see Fig. 8A-C and D-F, for an example of a melanoma metastasis see Fig. 8G-I). In 13 of 15 tumors virtually all melanoma cells stained SgII-positive whereas in two melanomas SgII-positive immunoreactions were enhanced or exclusively detectable in superficial tumor regions. Immunohistochemistry was conducted with two different rabbit antisera against SgII (GTX116446 and LS-C39034) which yielded identical results in the vast majority of tissue samples (Table S3).

When the same tumors were labelled with antibodies to Dsg2, all primary melanomas and 7 of 8 melanoma metastases exhibited rather diffuse Dsg2-positive immunoreactions within the cytoplasm and/or at the cell surface of the tumor cells (Fig. 8J-R; Table S3). By contrast, intercellular junctions between melanoma were mostly Dsg2-negative, except in two primary melanomas and one melanoma metastasis in which Dsg2-positive immunoreactions appeared to be focally enhanced at cell-cell contacts of 5-10% of the tumor cells (Fig. 8N and Q; Table S3).

Taken together, our immunohistochemical findings suggest (1) that SgII is widespread in malignant melanomas and their metastases, supporting the hypothesis that upregulation of SgII may be relevant for their pathogenesis *in vivo*, and (2) that Dsg2 is not junction bound but atypically distributed in most melanomas and melanoma metastases.

## Discussion

Our study provides evidence that depletion of Dsg2 leads to increased migration of melanoma cells rich in endogenous Dsg2. Increased migration upon Dsg2 depletion was also observed in cells pretreated with mitomycin C, indicating that this phenomenon occurs independent of proliferation. Furthermore, gene expression profiling data suggested a correlation between Dsg2 depletion and increased expression of migration-related genes.

In Matrigel-coated Transwell assays we additionally documented increased invasion of Dsg2-depleted melanoma cells. However, this effect may be mainly attributable to enhanced migration. To evaluate the impact of Dsg2 on invasive properties of melanoma cells independent of their migratory capacities, we used a TEER breakdown assay, which allows to measure cancer cell-monolayer violation at the very beginning of invasion with a very high sensitivity [26]–[29]. In this assay we did not find any differences between Dsg2-depleted and control cells.

It is well conceivable that Dsg2-related migration activities are in part due to altered cell adhesion. In epithelial cells, reduction or blockade of Dsg2 weakens intercellular adhesion whereas increased Dsg2 expression was demonstrated to promote adhesion [38]–[40]. However, in keratinocytes this protein is less important for cohesion than Dsg3 [41]. Dsg2 was recently reported to associate with caveolin-1, a major component of caveolae, and this association was discussed as a mechanism for regulating mitogenic signaling and cell surface presentation of Dsg2 [42]. How exactly the cell surface expression of Dsg2 is modulated in melanoma cells and how the protein alters adhesion of these cells remains to be studied.

Although the best established function of Dsg2 is to mediate cell-cell adhesion, it has emerged that the protein exerts further important functions [43]. Dsg2 was recently identified as the primary high-affinity receptor for serotype B adenoviruses, which cause respiratory and urinary tract infections [44]. Binding of these adenoviruses to Dsg2 leads to opening of junctions and to events reminiscent of epithelial-to-mesenchymal transition [44]. Recently, a small recombinant molecule derived from the sequence of adenovirus 3, termed “Junction Opener 1” (JO-1), was developed that mediates cleavage of Dsg2 dimers in epithelial cells [45]. Application of JO-1 was shown to increase penetration of monoclonal antibodies and chemotherapeutics into epithelial cancers *in vitro* and in animal models [45], [46]. Therefore, this molecule is discussed as a novel co-therapy for treatment of Dsg2-rich cancers, a concept that might also be applicable for the subset of Dsg2-containing malignant melanomas.

Furthermore, Dsg2 was implicated in apoptosis [47]–[49]. We did not systematically assess apoptosis, but Dsg2-depleted cells and controls appeared equally viable in all experiments. Moreover, cell culture supernatants of Dsg2-depleted cells and controls contained similar levels of lactate dehydrogenase, demonstrating no changes in membrane integrity and therefore unchanged viability (data not shown).

Dsg2-depleted embryonic stem cells display impaired proliferation, leading to death at or shortly after implantation [50]. Correspondingly, ectopic expression of Dsg2 in suprabasal keratinocytes causes hyperproliferation of the epidermis and development of skin tumors [51]. By contrast, neither our BrdU incorporation experiments nor our MTT assays supported involvement of Dsg2 in melanoma cell proliferation. These discrepancies may indicate that the impact of Dsg2 on proliferation is cell type- or context-dependent, all the more as distribution and complex formation of Dsg2 in epithelial cells and melanoma cells is completely different.

Alterations in Dsg2 were documented in a number of tumors. Dependent on the specific kind of tumor, the protein may be up- or downregulated. Basal cell carcinomas, squamous carcinomas and prostate carcinoma cells with high metastatic potential contain increased amounts of Dsg2 [52]–[54] whereas in certain types of gastric carcinomas Dsg2 is reduced or abnormally distributed [55], [56]. We have detected Dsg2 in two of eight melanoma cell lines in culture [21] and in a subset of human melanomas and metastases. In cultured melanoma cells Dsg2 is distributed diffusely on the cell surface, as previously demonstrated by confocal laser scanning and immunoelectron microscopy [21]. In the majority of primary melanomas and melanoma metastases analyzed here we noted rather diffuse Dsg2 immunoreactions in the cytoplasm and/or on the cell surface. Concentration of Dsg2 at cell boundaries was observed only rarely and only focally in small clusters of melanoma cells. This finding is in line with our previous observations [57] and with observations of another group [54], according to which expression of Dsg2 at intercellular junctions of melanomas *in vivo* is rare. Of course localization patterns detected by immunostaining strongly depend on the methods and reagents applied, and of course occurrence of Dsg2 in melanomas *in vivo* will have to be confirmed by different techniques including quantitative PCR and Western blot. Moreover the exact subcellular distribution of Dsg2 in these tumors remains to be uncovered, all the more as they do not contain desmosomes or desmosome-like structures, and the prognostic value of Dsg2 in melanomas remains to be determined.

Noteworthy, we observed increased expression of SgII and its bioactive peptide SN upon Dsg2 depletion. SgII is a member of the granin family of secretory peptides which is widely distributed in the nervous, endocrine and immune system [33], [37]. It is proteolytically processed into several bioactive peptides, most importantly, the 33-aa peptide SN (hSCGII_182-204_; SN; [31]) which plays an important role in neurogenic inflammation, chemotaxis and transendothelial migration. SN was demonstrated to stimulate migration of fibroblasts, smooth muscle cells and endothelial cells [34]–[36] and to attract diverse kinds of immune cells, including monocytes, immature dendritic cells and eosinophils [58]–[61]. It promotes transendothelial migration of leucocytes by reducing the expression of the tight junction proteins occludin and Zonula occludens-1 [62], [63]. Moreover, SN can directly trigger angio- and vasculogenesis by increasing the binding of vascular endothelial growth factor to endothelial cells [64] and activating PI3K/Akt and MAPK pathways [65], [66]. The contents of SN found in our Dsg2-depleted MeWo and C32 cells are relatively low compared to SN amounts in neural, endocrine and immune cells, however comparable to those found in smooth muscle tissue where SN is induced under ischemic conditions [67], [68]. Our findings suggest that overproduction of SN upon Dsg2 depletion may represent a novel mechanism triggering melanoma cell migration. On the one hand, it is conceivable that melanoma cells stimulate themselves in an autocrine manner. On the other, melanoma cells *in vivo* may be stimulated and attracted by SN secreted from other cell types, such as immune cells. Moreover, it is conceivable that SN promotes transendothelial migration of melanoma cells during metastasis and angiogenesis within melanomas, conferring to enhanced tumor growth.

Clearly, regulation and processing of SgII and SN in melanoma cells will have to be examined in more detail in further studies. Moreover, it will be interesting to study the occurrence, the amounts and the regulation of SgII and SN in other melanoma cell lines, including Dsg2-negative ones, and to correlate findings to their migratory capacity.

SgII and its splice product SN have never been reported before in malignant melanomas. We have demonstrated here for the first time that SgII is expressed both in cultured melanoma cells and in melanomas and melanoma metastases *in vivo*. Of course our immunohistochemical findings will have to be confirmed in a larger sample of melanomas and metastases. The exact role of SgII and SN in malignant melanomas and their potential prognostic value remain to be determined. However, our findings suggest that SgII is widespread in these tumors.

In conclusion our data add a new pathway of regulating melanoma cell migration related to a newly described Dsg2 – SgII axis. Upregulation of SgII may be a frequent event in melanomas contributing to increased melanoma cell migration and thereby to tumor progression.

## Supporting Information

Table S1 **Biological processes and pathways associated with Dsg2 depletion of C32 cells.** Genes found to be more than 1.5-fold up- or downregulated in Dsg2 siRNA-treated compared to non-targeting siRNA-treated C32 cells were analyzed for involvement in biological processes. “Total genes” indicates the total number of genes associated with the respective process that are contained in the expression array. “Changed genes” indicates the number of genes that are >1.5-fold up- or downregulated upon Dsg2 depletion. The significance level of enrichment is given as Log 10 (p). Significant association between Dsg2 depletion and a process or pathway is assumed when false discovery rates (FDR) are ≤0.05. MAPK – mitogen-activated protein kinase; TGF-β – transforming growth factor beta.
(DOC)

Table S2 **Gene expression profiles of Dsg2-depleted MeWo and C32 compared to controls: combined results.** Genes >1.5-fold up- or downregulated both in Dsg2-depleted compared to non-targeting siRNA-treated C32 and in Dsg2-depleted MeWo compared to MeWo controls were categorized according to biological processes. To calculate enrichment, the number of total genes involved in the respective process contained in the array was compared to the number of genes that were >1.5-fold differentially regulated upon Dsg2 deletion. Significance levels of enrichment are indicated as Log 10 (p). False discovery rates were insignificant (not shown). MAPK – mitogen-activated protein kinase.
(DOC)

Table S3 **Immunohistochemistry with SgII and Dsg2 antibodies on paraffin sections of primary melanomas and melanoma metastases.** Antibodies to SgII displayed cytoplasmic and sometimes granular immunoreactions. Dsg2 antibodies reacted diffusely in the cytoplasm and/or at the cell surface. In addition, some tumors exhibited focal Dsg2-positive cell border staining. Immunoreactions were classified as negative (neg.), weakly positive (+), positive (++) or strongly positive (+++). The percentage of immunoreactive melanoma cells within each tumor was determined in 10 optical fields at 100-fold magnification. Tumor thickness according to Breslow is indicated in µm. Grading was performed according to the American Joint Committee on Cancer 2009 classification. NMM – nodular malignant melanoma; SSM – superficial spreading melanoma.
(DOC)
