# Supplementary material for: Desmoglein 2 Depletion Leads to Increased Migration and Upregulation of the Chemoattractant Secretoneurin in Melanoma Cells
Source: PLoS One. 2014 Feb 18;9(2):e89491. doi: 10.1371/journal.pone.0089491 (PMC3928442; doi:10.1371/journal.pone.0089491)
Supplement: Table S1 — Biological processes and pathways associated with Dsg2 depletion of C32 cells. Genes found to be more than 1.5-fold up- or downregulated in Dsg2 siRNA-treated compared to non-targeting siRNA-treated C32 cells were analyzed for involvement in biological processes. “Total genes” indicates the total number of genes associated with the respective process that are contained in the expression array. “Changed genes” indicates the number of genes that are >1.5-fold up- or downregulated upon Dsg2 depletion. The significance level of enrichment is given as Log 10 (p). Significant association between Dsg2 depletion and a process or pathway is assumed when false discovery rates (FDR) are ≤0.05. MAPK – mitogen-activated protein kinase; TGF-β – transforming growth factor beta. (DOC) [file pone.0089491.s001.doc]

**Supplementary Table S1. Biological processes and pathways associated with Dsg2 depletion of C32 cells**

| **Biological process** | **Total genes** | **Changed genes** | **Enrichment** | **Log 10 (p)** | **FDR** |
| --- | --- | --- | --- | --- | --- |
| **Anatomical structure morphogenesis** | 1062 | 39 | 2.161419 | -5.445155 | 0.010000 |
| **Cell adhesion** | 761 | 29 | 2.285151 | -4.568621 | 0.003333 |
| **Cell migration** | 333 | 16 | 2.881226 | -3.823661 | 0.020000 |
| **Cell motility** | 369 | 17 | 2.762639 | -3.810164 | 0.021429 |
| **Developmental process** | 3260 | 77 | 1.416363 | -3.376481 | 0.041333 |
| **Protein kinase cascade** | 480 | 19 | 2.373635 | -3.353637 | 0.037059 |
| **MAPK cascade** | 204 | 11 | 3.233435 | -3.198238 | 0.041500 |
| **Response to lipid** | 8 | 3 | 22.487069 | -3.617997 | 0.036250 |
| **Response to extracellular stimulus** | 134 | 9 | 4.027535 | -3.387762 | 0.044286 |
| **TGF-β receptor signaling pathway** | 109 | 8 | 4.401139 | -3.324072 | 0.036111 |
| **Transmembrane receptor protein serine threonine kinase signaling pathway** | 135 | 9 | 3.997701 | -3.364028 | 0.038750 |
