# Supplementary material for: Desmoglein 2 Depletion Leads to Increased Migration and Upregulation of the Chemoattractant Secretoneurin in Melanoma Cells
Source: PLoS One. 2014 Feb 18;9(2):e89491. doi: 10.1371/journal.pone.0089491 (PMC3928442; doi:10.1371/journal.pone.0089491)
Supplement: Table S2 — Gene expression profiles of Dsg2-depleted MeWo and C32 compared to controls: combined results. Genes >1.5-fold up- or downregulated both in Dsg2-depleted compared to non-targeting siRNA-treated C32 and in Dsg2-depleted MeWo compared to MeWo controls were categorized according to biological processes. To calculate enrichment, the number of total genes involved in the respective process contained in the array was compared to the number of genes that were >1.5-fold differentially regulated upon Dsg2 deletion. Significance levels of enrichment are indicated as Log 10 (p). False discovery rates were insignificant (not shown). MAPK – mitogen-activated protein kinase. (DOC) [file pone.0089491.s002.doc]

**Supplementary Table S2. Gene expression profiles of Dsg2-depleted MeWo and C32 cells compared to controls: combined results.**

| **Biological process** | **Enrichment** | **Log 10 (p)** | **Genes upregulated upon Dsg2 depletion** | **Genes downregulated upon Dsg2 depletion** |
| --- | --- | --- | --- | --- |
| **Cell motility** | 4.308788 | -1.875223 | HMOX1, LAMC1, SCG2 | ROPN1B |
| **Protein kinase cascade** | 4.968571 | -2.945959 | GOLT1B, HMOX1, MDFIC, SCG2, STRADB | TIMP2 |
| **MAPK cascade** | 7.793838 | -2.783447 | MDFIC, SCG2, STRADB | TIMP2 |
| **Phosphate metabolic process** | 2.390378 | -1.623518 | CDK6, MDFIC, MTMR6, SAPS3, STRADB | ATP6V1C1, MAPK6 |
| **Regulation of proliferation** | 3.559574 | -2.222388 | CDK6, HMOX1, LAMC1, SCG2 | SPARC, TIMP2 |
| **Cell development** | 2.876163 | -1.550769 | CDK6, LAMC1, MBNL1 | TIMP2, DCT |
| **Negative regulation of apoptosis** | 3.761694 | -1.348207 | HMOX1, SCG2, STRADB |  |
| **Others** |  |  | CASP7, IGF2BP3, LAMP2, LMNB1, MED20, MTAP, PDE7B, PLSCR4, RNF144A, TROVE2, UBE2E3, WDFY1 | AGTRAP, C9ORF69, C17ORF58, C20ORF108, COG6, DBNDD1, DSG2, DYNC1/1, FYCO1, HDHD2, IGSF3, INPP5A, NCRNA00094, PI15, TBC1D9, TMEM97, ZFYVE21 |
