# Supplementary material for: Desmoglein 2 Depletion Leads to Increased Migration and Upregulation of the Chemoattractant Secretoneurin in Melanoma Cells
Source: PLoS One. 2014 Feb 18;9(2):e89491. doi: 10.1371/journal.pone.0089491 (PMC3928442; doi:10.1371/journal.pone.0089491)
Supplement: Table S3 — Immunohistochemistry with SgII and Dsg2 antibodies on paraffin sections of primary melanomas and melanoma metastases. Antibodies to SgII displayed cytoplasmic and sometimes granular immunoreactions. Dsg2 antibodies reacted diffusely in the cytoplasm and/or at the cell surface. In addition, some tumors exhibited focal Dsg2-positive cell border staining. Immunoreactions were classified as negative (neg.), weakly positive (+), positive (++) or strongly positive (+++). The percentage of immunoreactive melanoma cells within each tumor was determined in 10 optical fields at 100-fold magnification. Tumor thickness according to Breslow is indicated in µm. Grading was performed according to the American Joint Committee on Cancer 2009 classification. NMM – nodular malignant melanoma; SSM – superficial spreading melanoma. (DOC) [file pone.0089491.s003.doc]

**Supplementary Table S3. Immunohistochemistry with SgII and Dsg2 antibodies on paraffin sections of primary melanomas and melanoma metastases**

| **No.** | **Kind of tumor** | **SgII GTX116446** | **SgII LS-C39034** | **Dsg2 rb5** |
| --- | --- | --- | --- | --- |
| 1 | Primary NMM, 10 mm, pT4a | ++, 100% | ++, 100% | + diffusely, 100%  Focally enhanced at cell-cell junctions, ca. 10% |
| 2 | Primary NMM, 3.9 mm, pT3a | ++, 100% | ++, 100% | + diffusely, 100% |
| 3 | Primary SSM, 0.95 mm, ulcerated, pT1b | +++, 100% | ++, 100% | + diffusely , 100% |
| 4 | Primary NMM, 0.8 mm, pT1a | +++, 100% | ++, 100% | ++ diffusely, 100% |
| 5 | Primary SSM, 1.05 mm, ulcerated, pT2b | ++ in superficial tumor areas, ca. 50%  neg. in deeper areas | ++ in superficial tumor areas, ca. 50%  neg. in deeper areas | + diffusely in super-ficial areas, ca. 30%  neg. in deeper areas |
| 6 | Primary SSM, 0.7 mm, pT1a | ++, 100% | ++, 100% | + diffusely, 100% |
| 7 | Primary mucosal melanoma of the anus | ++, 100% | ++, 100% | + diffusely, ca. 50%  Focally enhanced at cell-cell junctions, ca. 5% |
| 8 | Melanoma metastasis, breast | ++, 100% | ++, 100% | + diffusely, 100% |
| 9 | Cutaneous metastasis, trunk | +++, 100% | ++, 100% | + diffusely, 100% |
| 10 | Lymph node metastasis | ++, 100% | ++, 100% | + diffusely, 100% |
| 11 | Intramuscular metastasis | ++, 100% | ++, 100% | neg. |
| 12 | Cutaneous metastasis, chin | ++, 100% | ++, 100% | ++ diffusely, 100% |
| 13 | Metastasis of the small intestine | +, 100% | +, 100% | + diffusely, 100% |
| 14 | Cutaneous metastasis, foot sole | + in superficial tumor regions, ca. 50%  neg. in deeper regions | +, 100%, reactivity enhanced in superficial tumor regions | + diffusely, 100% |
| 15 | Cutaneous metastasis, temple | ++, 100% | ++, 100% | ++ diffusely, 100%,  Focally enhanced at cell-cell junctions, ca. 10% |
